# Brain Cortical Complexity and Subcortical Morphometrics in Lifelong Premature Ejaculation

**DOI:** 10.3389/fnhum.2020.00283

**Published:** 2020-07-22

**Authors:** Jiaming Lu, Lihua Yuan, Jiaxuan Jin, Shangwen Yang, Wen Zhang, Ming Li, Xin Zhang, Junxia Wang, Sichu Wu, Qian Chen, Zhao Qing, Yutian Dai, Bing Zhang, Zhishun Wang

**Affiliations:** ^1^Department of Radiology, The Affiliated Drum Tower Hospital of Nanjing University Medical School, Nanjing, China; ^2^Department of Radiology, Nanjing Drum Tower Hospital Clinical College of Nanjing Medical University, Nanjing, China; ^3^Institute of Brain Science, Nanjing University, Nanjing, China; ^4^Department of Andrology, The Affiliated Drum Tower Hospital of Nanjing University Medical School, Nanjing, China; ^5^Columbia University Vagelos College of Physicians and Surgeons, New York, NY, United States

**Keywords:** premature ejaculation, morphology, vertex analysis, gyrification index, mediation analysis

## Abstract

Premature ejaculation (PE) is the most common male sexual dysfunction. The brain disturbances that cause this disorder remain poorly understood. This study aimed to investigate how the morphology of cortical and subcortical brain structures differed in PE, how these morphologic differences were associated with severity measures of PE, such as intravaginal ejaculatory latency time (IELT), and how these cortical and subcortical structures were causally connected through mediation analysis. Anatomical MRI scans were acquired from 39 male participants, 23 with PE (28.78 ± 4.32 years), and 16 without PE (27.88 ± 3.65 years). We used a subcortical analysis package within FSL to perform subcortical shape segmentation and statistical analysis. The PE group was compared with the normal control (NC) group in the shapes of 15 subcortical structures with general linear models [*p* < 0.05, family-wise error (FWE)-corrected]. We analyzed the cortical complexity revealed by the gyrification index using the Computational Anatomy Toolbox (CAT12). Vertex-wise shape analyses revealed outward shape deformations (expansions) in the left hippocampus and bilateral thalamus. Gyrification index analyses revealed that the right orbital frontal cortex and the right nucleus accumbens had greater complexity in PE patients. The shape deformations were positively correlated with the IELTs in the NC group, while this relationship was interrupted in the PE group. PE is associated with outward deformations of the subcortical surfaces and more complexity of the cortical structures. These morphological differences may be the basis of the brain functional alterations underlying PE.

## Introduction

Ejaculation is the final stage of coitus in mammalian males and is mandatory for natural procreation (Clement and Giuliano, 2016). Affecting approximately 21% to 30% of the population, premature ejaculation (PE) is one of the most common sexual dysfunctions (Laumann et al., 2005; Gur and Sikka, 2015; Saitz and Serefoglu, 2015), reducing the quality of sexual life of the individuals and their partners (Porst et al., 2007). Based on the 2014 International Society of Sexual Medicine (ISSM) definition, PE can be classified as either lifelong PE or acquired PE (Serefoglu et al., 2014a). Ejaculation that always or nearly occurs before or within approximately 1 min of vaginal penetration from the first sexual experience is defined as lifelong PE, and a clinically significant decrease in latency time, often no longer than approximately 3 min, is defined as acquired PE. PE may be commonly underreported because of embarrassment and a lack of awareness. Also, many psychiatric disorders, such as anorgasmia and low libido, as well as depression and anxiety, are comorbidities significantly associated with PE (Waldinger et al., 2009).

Although PE is highly prevalent, it has been difficult to understand its mechanism. The PE pathology has been reported to be associated with depression and anxiety, and one possible therapeutic strategy is SSRI administration. Innate (genetic) abnormalities such as 5-HTT promotor region polymorphism have been reported previously (Janssen et al., 2009). Brain serotonin is responsible for sexual inhibition, and the serotonin and DA systems are interconnected and mutually inhibitory concerning sexual behavior (Pfaus, 2009). By utilizing non-invasive instruments such as magnetic resonance imaging (MRI), we can objectively assess the brain function of individuals with PE during ejaculation, leading to a better understanding of sexual dysfunction in men. Previous functional MRI (fMRI) studies have found that under erotic visual stimulation, PE patients showed significant activation in the orbital frontal cortex (OFC), anterior cingulate gyrus, insula, amygdala, and septal areas (Mouras et al., 2003; Kim et al., 2006; Mallick et al., 2007; Zhang et al., 2017). Additionally, compared to that of controls, functional connectivity is significantly reduced within the dopamine pathway areas of individuals with PE (Lu et al., 2018).

In the present study, we hypothesized that, compared with normal controls (NCs), the PE group would exhibit structural alterations (larger and more complex) in cortical and subcortical regions. These changes may influence the functional performance of individuals with PE.

## Materials and Methods

### Participants

Twenty-three right-hand–dominant patients with lifelong PE and 16 right-hand–dominant NCs were enrolled in this study from 2012–2014 in Nanjing Drum Tower Hospital. The details of the subjects can be found in our previous publications (Zhang et al., 2017; Lu et al., 2018). Four subjects (three PE patients and one NC) were not included in this study because they only underwent a structure scan. Lifelong PE patients were diagnosed according to the ISSM guidelines (Serefoglu et al., 2014a): (a) ejaculation that always or nearly always occurs before or within approximately 1 min of vaginal penetration; (b) the inability to delay ejaculation on all or nearly all vaginal penetrations; and (c) negative personal consequences such as distress, bother, frustration, and/or the avoidance of sexual intimacy. NCs were recruited with self-reported intravaginal ejaculatory latency time (IELT) of more than 3 min. The IELTs were averaged from a 4-week baseline period during which both patients and NCs were asked to have sexual intercourse at least four times. Patients with erectile dysfunction [International Index of Erectile Function (IIEF)-5 score <21], reduced sexual desire or inhibited male orgasm were excluded from the study. Moreover, patients with mental disorders, physical illnesses that affect ejaculatory function, abuse of alcohol, and any medical treatment for PE in the past six months were also excluded. The following two questionnaires were conducted for all participants: the IIEF-5 (Rhoden et al., 2002) and the Chinese Index of Sexual Function for Premature Ejaculation (CIPE)-5 (Yuan et al., 2004). The CIPE-5 includes five items: the IELT; difficulty in prolonging sexual intercourse; sexual satisfaction; partner’s sexual satisfaction; and frequency of feeling anxious, depressed, or stressed during sexual activity. Each item was designed to be addressed by the subject on a 5-point Likert-type scale, and the score of each item was added to generate a total score. This study was performed according to the Declaration of Helsinki and approved by the institutional review boards of the Nanjing Drum Tower Hospital. Written informed consent was obtained from each subject.

### Image Acquisition

Imaging was performed on a 3 T Achieva TX MRI system with an 8-channel head coil and SPGR pulse sequence. The detailed acquisition parameters of the high-resolution 3D T1-weighted brain structural images were as follows: *TR* = 7,600 ms; *TE* = 3,400 ms; flip angle = 8°; *FOV* = 256 × 256 × 192 mm^3^ and slice thickness = 1 mm.

### Subcortical Shape Analyses

We first pre-processed each anatomical image using the DARTEL algorithm from the VBM toolbox (run in Statistical Parametric Mapping software (SPM12^1^) to perform intensity correction and skull stripping. A Bayesian model-based segmentation toolbox in FSL (FIRST^2^) was used to segment each anatomical image and create vertex meshes for 15 subcortical structures (brain stem and bilateral nucleus accumbens, putamen, caudate, palladium, thalamus, hippocampus, and amygdala). Quality control of the subcortical segmentation was performed by an experienced image analyst, the first author, following FSL FIRST guidelines^3^. No participants were excluded because of poor segmentation of one or more structures. For each participant and subcortical structure, a vertex index was calculated using the FSL vertex analysis script *first_utils* script, which was based on the signed perpendicular distance from the surface mesh (a vtk file generated by using FSL *run_first_all* script) of that corresponding structure in the Montreal Neurological Institute (MNI) template. Positive indices represented *outward deformations* or expansion of the surface of a given structure, and negative indices represented *inward deformations* or contraction (atrophy) of that structure’s surface. These values were then submitted to statistical analysis.

### Cortical Gyrification Index (GI) Analysis

The GI analysis was conducted using the Computational Anatomy Toolbox (CAT12^4^), which is an extensive toolbox of SPM12. The default settings described in the CAT12 toolbox were adopted in this study. CAT12 provides a fully automated method to estimate the central surface of the hemispheres based on the projection-based thickness method (Dahnke et al., 2013). The GIs were extracted from central surface data based on the absolute mean curvature as previously described (Luders et al., 2006). The GI images of the left and right hemispheres were smoothed with a 20-mm full-width at half-maximum (FWHM) Gaussian kernel.

### Statistical Analyses

Group differences in subcortical shape were assessed using nonparametric method-based group comparisons for each of the 15 subcortical structures using the FSL *randomize* procedure and included age as a covariate. We report findings corrected for multiple comparisons using threshold-free cluster enhancement (TFCE) with a family-wise error (FWE) rate of *p* < 0.05 by running 5,000 random permutations. We used a supercomputer, high-performance computing (HPC) cluster to run statistical jobs in a parallel mode controlled by Sun Grid Engine (SGE).

## Results

### Characteristics of the Study Cohort

Anatomical MRI data from the 39 participants were included in the analyses, including 23 PE patients and 16 NCs. The participant characteristics are detailed in Table 1. There were no significant differences in age, education level, or marital status. All the patients with PE and the controls had normal erectile function (the normal score range of the IIEF-5 was between 22 and 25; Rosen et al., 1999). The IELTs were significantly different between the PE and NC groups. Detailed information can be found in our previous work (Zhang et al., 2017; Lu et al., 2018).

**Table 1:** Demographic and clinical characteristics of patients and control participants.

	PE (*n* = 23)	NC (*n* = 16)	*p*
Age (years)			
*Mean ± SD*	27.80 ± 4.30	27.90 ± 3.70	0.945
Marital status (%)			0.743^#^
*Single/Married*	47.82/52.18	37.50/62.50	
Education level (%)			0.916^#^
*Elementary*	17.39	12.50	
*High school*	34.78	37.50	
*University*	47.83	50.00	
IIEF-5 score	23.83 ± 1.23	24.13 ± 0.72	
*Mean ± SD*	23.75 ± 1.25	24.13 ± 0.74	0.389
CIPE			
*Q1*	1.90 ± 0.70	5.00 ± 0.00	<0.01
*Q2*	1.13 ± 0.34	4.06 ± 0.77	<0.01
*Q3*	1.22 ± 0.42	4.13 ± 0.72	<0.01
*Q4*	1.22 ± 0.42	4.13 ± 0.62	<0.01
*Q5*	3.30 ± 1.55	4.63 ± 0.50	<0.01
*Total*	8.78 ± 2.21	21.94 ± 2.02	<0.01
IELT (in min)			
*Mean ± SD*	0.83 ± 0.41	10.65 ± 6.61	<0.01

The data from questionnaires are presented in terms of the mean scores (Mean) and standard deviations (SD) in the premature ejaculation (PE) and normal control (NC) groups. IIEF-5, International Index of Erectile Function-5; CIPE-5, Chinese Index of Premature Ejaculation with 5 questions; IELT, intravaginal ejaculatory latency time. ^#^Chi-squared test.

### Group Differences in the Subcortical Brain Structures

**Figure 1:**
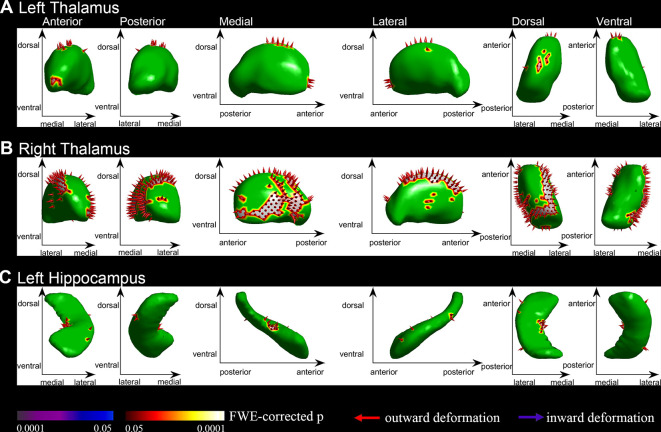
Vertex-wise shape analysis between premature ejaculation (PE) patients and normal controls (NCs). **(A)** Outward shape deformations (expansions) in the left thalamus. **(B)** Outward shape deformations (expansions) in right thalamus. **(C)** Outward shape deformations (expansions) in the left hippocampus. FWE-corrected, *p* < 0.05.

We segmented each brain into 15 subcortical brain structures including the brain stem, left and right nucleus accumbens, left and right putamen, left and right caudate, left and right palladium, left and right thalamus, left and right hippocampus and left and right amygdala. Group comparisons were performed on each of the 15 structures on the HPC cluster. Compared to the NC group, while regressing outage, the PE group had significant outward shape deformations (expansions) in the following subcortical structures (all *p* < 0.05, FWE-corrected with TFCE): left and right thalamus (Figures 1A,B, Table 2; *p* = 0.009 and *p* = 0.003, respectively) and left hippocampus (Figure 1C, Table 2; *p* = 0.001). No significant inward shape deformations (contractions) in subcortical structures were found.

**Table 2:** The group differences between patients and NCs in vertex analysis of subcortical regions.

	PE (*n* = 23)	NC (*n* = 16)	*t*	*p*
L_Hipp	0.09 ± 0.13	−0.05 ± 0.11	3.68	0.001
L_Thal	0.03 ± 0.10	−0.5 ± 0.11	2.75	0.009
R_Thal	0.04 ± 0.09	−0.06 ± 0.11	3.22	0.003

The data from the vertex analysis are presented in terms of the mean scores (Mean) and standard deviations (SD) in the premature ejaculation (PE) and normal control (NC) groups. L_Hipp, left hippocampus; L_Thal, left thalamus; R_Thal, right thalamus.

### Group Differences in the Cortical GI Analysis

**Figure 2:**
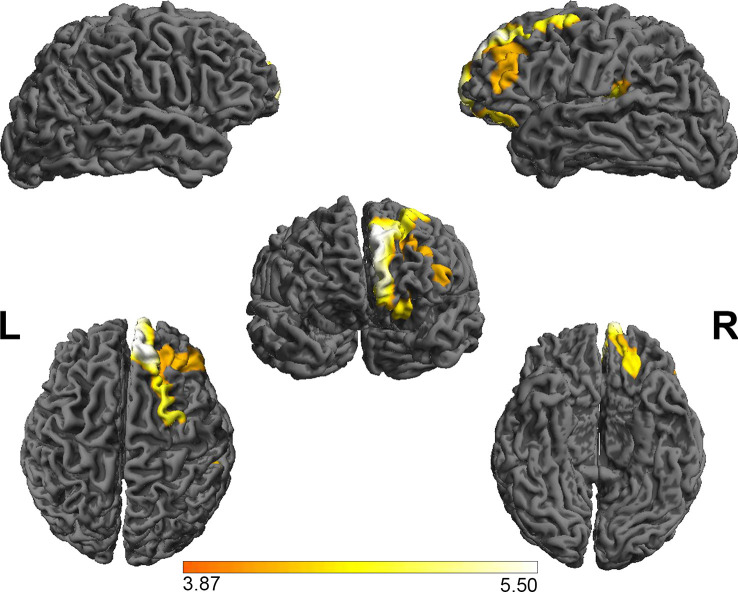
The group differences in the gyrification index between PE patients and NCs. The results showed that the gyrification index in the right OFC and the right nucleus accumbens in the PE group was greater than in the NC group. FWE-corrected, *p* < 0.05. OFC, orbital frontal cortex.

Based on the absolute mean central cortical surface curvature, the whole brain GI was compared between the PE group and the NC group. The PE group had significantly increased GI in the right OFC and right nucleus accumbens (*p* < 0.05, FWE-corrected with TFCE). Figure 2 shows the right OFC and the right nucleus accumbens where there were group differences. Table 3 shows the mean GI extracted from each subject in the brain areas with significant group differences. No significant cortical structures with decreased GI were found.

**Table 3:** The group differences between patients and NCs in the gyrification index.

	PE (*n* = 23)	NC (*n* = 16)	*t*	*p*
OFC	30.27 ± 0.63	29.14 ± 0.61	5.57	<0.001
Nucleus accumbens	29.88 ± 0.78	28.16 ± 1.04	5.89	<0.001

The data from the GI were presented in terms of the mean scores (Mean) and standard deviations (SD) in the premature ejaculation (PE) and normal control (NC) groups. OFC, orbital frontal cortex; GI, gyrification index.

### Correlation Between MRI Morphology and Clinical Measurements (CIPE-5 and IELT)

**Figure 3:**
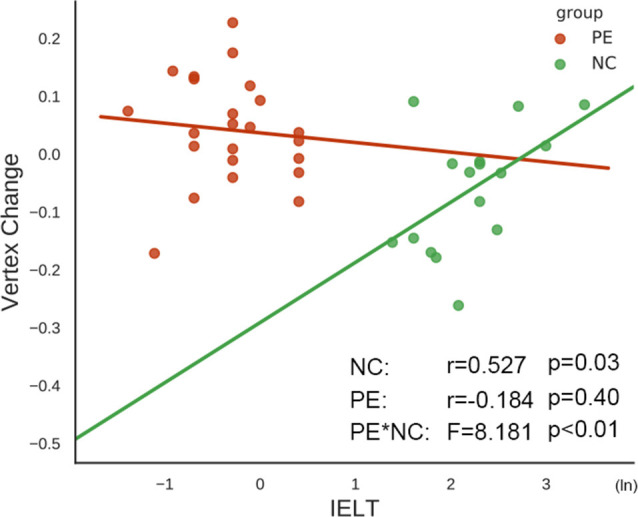
The correlations between the vertex changes in the right thalamus and the intravaginal ejaculatory latency time (IELTs). The vertex changes in the right thalamus were significantly positively correlated with the IELTs in the NC group, while there was no significant correlation in the PE group. The interaction effect of the correlation between the PE and NC groups was significant.

The IELT is a simple but important quantitative assessment used to evaluate PE. In our cohort, the subcortical vertex changes in the right thalamus were significantly positively correlated with the IELTs in the NC group (*r* = 0.527, *p* = 0.03), while there was no significant correlation in the PE group between the vertex changes and IELTs. The interaction effect for the correlation between the PE and NC groups was significant (*F* = 8.181, *p* < 0.01, Figure 3). However, there were no significant correlations between the GIs and IELTs in either the NC group or the PE group (*p* > 0.05). The correlation between MRI morphology and the CIPE-5 was the same as that for the IELT (NC: *r* = 0.563, *p* = 0.02; interaction effect: *F* = 9.63, *p* < 0.01).

## Discussion

This study examined the subcortical and cortical brain structure of participants who presented with PE. The subcortical shape analysis revealed that the PE group had a significant shape expansion in the left hippocampus and bilateral thalamus. Additionally, the PE group showed increased GI in the right OFC and nucleus accumbens. These subcortical and cortical brain regions constitute several very important neural circuits that are thought to be generally involved in controlling human memory and emotion and specifically play key roles in the memory and dopamine reward system. The morphological changes in the right thalamus were positively correlated with the IELT in the NC group, while no significant correlation was observed in the PE group.

In this study, we found a significant shape expansion in the bilateral thalamus. The thalamus has multiple functions and is generally believed to act as a relay station, or hub, relaying information between different subcortical areas and the cerebral cortex (Gazzaniga and Ivry, 2013). In particular, most sensory systems (excluding the olfactory system) include a thalamic nucleus that receives sensory signals and sends them to the associated primary cortical area. Additionally, the thalamus makes connections between the subcortical regions (e.g., connections to the hippocampus *via* the mammillothalamic tract) and the cerebral cortex (e.g., connections to the hippocampus *via* the thalamocortical radiations; Carlesimo et al., 2011). Our previous short functional connectivity density (SFCD) results showed that the thalamus had a decreased SFCD in PE patients compared to NCs (Lu et al., 2018). This may indicate that the functional change may have been caused by expanding structural changes in the thalamus. In other words, the larger the volume, the poorer the performance, which could not be explained by decompensation alone. This means that although the patients demonstrated enlarged structures, they were unable to maintain their previous performance. A variety of neurodegenerative disorders illustrate similar phenomena, such as an increased performance in MCI despite a thinner cortex (Cheng et al., 2018).

Several studies have demonstrated that functional and structural abnormalities of the subcortical structures are associated with affective processing, especially in the thalamus (Webb et al., 2014; Lu et al., 2016). The thalamus is a hub that transmits information from the spine to the cerebral cortex *via* the thalamus as a relay station. This information includes pain, temperature, itch, and crude touch, which are very important inputs for the sexual process (Georgiadis and Kringelbach, 2012; Saitz and Serefoglu, 2015). The thalamus is believed to process sensory information as well as relay it—each of the primary sensory relay areas receives strong feedback connections from the cerebral cortex. Our cortex shape analysis revealed that the right OFC and the right nucleus accumbens had an increased GI in the PE group. Gyrification is the process of forming the characteristic folds of the cerebral cortex (Rakic, 2009). The nucleus accumbens and OFC, key components in reward circuitry, have been suggested to be the pleasure generator (Heshmati and Russo, 2015; Loonen and Ivanova, 2016). The present study found increased complexity in the nucleus accumbens and OFC, which may make it easier for persons to prematurely orgasm during sexual intercourse and thus are at a greater risk for developing PE. These ideas are consistent with the findings in functional imaging studies. Previous studies also showed that the OFC and the nucleus accumbens had a decreased SFCD in the PE group (Lu et al., 2018). Additional studies have shown that there is an inverse relationship between cortical thickness and gyrification; areas of the brain with low values of thickness were found to have high levels of gyrification (Striedter et al., 2015). However, another study revealed that a larger GI is associated with a thicker cortex (Fornito et al., 2007). Although this remains a controversial finding, our cortical results may suggest that the subcortical and cortical structural changes may have led to functional changes, especially in the reward circuitry. Therefore, morphological changes in the brain may be the new insight into the foundation of PE pathology.

The MRI morphological changes were significantly positively correlated with the IELTs. The IELT is the time taken by a man to ejaculate during vaginal penetration (Waldinger et al., 2005) and is a criterion for diagnosing lifelong PE (Serefoglu et al., 2014b). The IELT may be relevant in the perception of sexual performance and actual satisfaction. We found that the vertex changes in the right thalamus were positively correlated with the IELTs in the NC group, while a negative trend was observed in the PE group, and the interaction effect between the slopes of the correlation was significant. The PE group had a larger mean cortex change in the right thalamus than the NC group overall, which may indicate that the brain tries to make up for a poor performance by expanding the shape of the thalamus. The worse the performance is, the greater the expansion of the shape required, which resulted in the negative trend between the shape in the right thalamus and the IELT in the PE group observed in this study. These findings may indicate a relationship between disorder severity and MRI morphology metrics, which could lead to a better understanding of PE using simple, accessible tools. Furthermore, by mediation analysis, we found that the subcortical shape change that affected the PE patients’ behavior was mediated by the cortical structure complexity.

The main limitation of this study was the self-reported IELTs, as mentioned in our previous studies (Zhang et al., 2017; Lu et al., 2018). The IELT was measured in the 4-week baseline period during which both patients and NCs were asked to have sexual intercourse at least four times to reduce the error. A stopwatch technique, i.e., using a timer, should obtain more accurate IELTs compared with self-report. In future studies, we should encourage the subjects to use stopwatch technology. The sample size of this study was small, and more subjects should be included to reflect the entire PE population. Moreover, detailed data on the clinical and demographical characteristics of the participants should be obtained. It would also be advisable to have complete questionnaires from patients with PE rather than to rely on self-reporting techniques to exclude possible confounding factors, which might include psychological factors such as the degree of the emotional attachment to the sexual partner as well as physical factors such as the sensitivity of genital organ itself. Finally, a follow-up study should be conducted to verify that these MRI morphological changes can be revised or altered with clinical intervention.

In conclusion, this study demonstrated that PE patients have undergone expanding subcortical deformations and presented greater complexity in cortical structures. The subcortical shape change was associated with PE-related behavior, and this association was mediated by the cortical structure complexity. It is believed that these morphological findings may enhance the understanding of PE-related brain functional changes and will provide new insight into this medical issue for the development of new therapeutic strategies, such as thalamus-targeting drugs.

## Data Availability Statement

The datasets generated for this study are available on request to the corresponding author.

## Ethics Statement

The studies involving human participants were reviewed and approved by Nanjing Drum Tower Hospital. The patients/participants provided their written informed consent to participate in this study.

## Author Contributions

JL, BZ, and ZW designed the study. JL wrote the manuscript. JL, LY, JJ, SY, WZ, ML, XZ, and JW performed the data management, data analysis, and figure creation. JL, QC and YD determined the structure and logic of the manuscript. ZQ and SW improved the language in the manuscript.

## Conflict of Interest

The authors declare that the research was conducted in the absence of any commercial or financial relationships that could be construed as a potential conflict of interest.

## Footnotes

^1^http://www.fil.ion.ucl.ac.uk/spm/software/spm12

^2^http://fsl.fmrib.ox.ac.uk/fsl/fslwiki/FIRST

^3^https://fsl.fmrib.ox.ac.uk/fsl/fslwiki/FIRST/UserGuide

^4^http://dbm.neuro.uni-jena.de/cat/
